# Effect of a single acupuncture treatment on surgical wound healing in dogs: a randomized, single blinded, controlled pilot study

**DOI:** 10.1186/1751-0147-52-57

**Published:** 2010-10-15

**Authors:** Erja E Saarto, Anna K Hielm-Björkman, Khadije Hette, Erja K Kuusela, Cláudia Valéria S Brandão, Stélio PL Luna

**Affiliations:** 1 Faculty of Veterinary Medicine, Department of Equine and Small Animal Medicine, P.O. Box 57, FI-00014 University of Helsinki, Finland, Europe; 2 Pieneläinvastaanotto, Torniomäentie 30, 45120 Kouvola, Finland, Europe; 3 Department of Veterinary Surgery and Anaesthesiology of the School of Veterinary Medicine and Animal Science of São Paulo State University, Brazil

## Abstract

**Background:**

The aim of the study was to investigate the effect of acupuncture on wound healing after soft tissue or orthopaedic surgery in dogs.

**Methods:**

29 dogs were submitted to soft tissue and/or orthopaedic surgeries. Five dogs had two surgical wounds each, so there were totally 34 wounds in the study. All owners received instructions for post operative care as well as antibiotic and pain treatment. The dogs were randomly assigned to treatment or control groups. Treated dogs received one dry needle acupuncture treatment right after surgery and the control group received no such treatment. A veterinary surgeon that was blinded to the treatment, evaluated the wounds at three and seven days after surgery in regard to oedema (scale 0-3), scabs (yes/no), exudate (yes/no), hematoma (yes/no), dermatitis (yes/no), and aspect of the wound (dry/humid).

**Results:**

There was no significant difference between the treatment and control groups in the variables evaluated three and seven days after surgery. However, oedema reduced significantly in the group treated with acupuncture at seven days compared to three days after surgery, possibly due the fact that there was more oedema in the treatment group at day three (although this difference was nor significant between groups).

**Conclusions:**

The use of a single acupuncture treatment right after surgery in dogs did not appear to have any beneficial effects in surgical wound healing.

## Background

The aim of wound healing is to promote rapid wound closure and prevent excess scar formation. Inflammation is the primary reaction at a wound site [1,2], followed by cellular proliferation, extra cellular matrix syntheses, remodelling and scar formation [2]. Cytokines, platelets, macrophages, neutrophils and monocytes all play an important role in the wound healing process [2]. Decreased blood flow to the wound bed increases the risk of infection and delays healing [3]. Surgical technique, experience of the surgeon, infection, mechanical stress of the wound, use of abrasive or inflammatory suture material, and radiation injury are other local factors that influence surgical wound healing [3]. Hyponatremia, hypovolemia, oedema, poor nutrition, vitamin deficiency, administration of corticosteroids, diabetes mellitus, administration of cytotoxic drugs, jaundice, uraemia and advanced age are systemic factors that influence wound healing [3]. In this context, the use of a single acupuncture session has been suggested as to provide a non-toxic and easy alternative to improve surgical wound healing in dogs.

Acupuncture is the insertion of needles into specific locations of the body, known as acupuncture points, for the treatment or prevention of many different diseases. The most common acupuncture technique is the so called "dry needle" acupuncture, where metal acupuncture needles are introduced into acupuncture points and left *in situ *for five to 60 minutes. Other forms of acupuncture point stimulations include electroacupuncture, laser, moxa, injections with different solutions, and permanent implants of gold or other materials [4,5].

Acupuncture relieves inflammation by different mechanisms [6-13]; increases blood circulation in the affected area, with subsequent increase of neuropeptides, cytokines and other vasoactive substances [1] as well as reduces oedema [8,12,14]. Acupuncture enhances wound healing accelerators such as fibroblast growth factors (FGF) and platelet-derived growth factors (PDGF) in experimental models [15,16]. It also increases the migration of neutrophiles and decreased the amount of local bacterias in experimentally-induced peritonitis in rats [6].

An acupuncture-like treatment improves the wound healing of chronic wounds in men [17] and electro-acupuncture improved the healing of chronic wounds in experimental animals not responsive to conventional treatment [18]. Acupuncture performed in acupuncture points GV14, GV2 and LIV13 reduced the rate of necrosis and improved the survival of dorsal skin flaps in rats [19]. To our knowledge there are no published studies about the effect of acupuncture for surgical wound healing in dogs.

Postoperative poor wound healing is a complication producing pain and discomfort, possible wound infection with need of prolonged use of antibiotics and sometimes even resulting in systemic symptoms. Resistance to antibiotics is a wound complication which has become progressively important due to easily spreading hospital epidemics. The aim of this study was to investigate the effect of an easily performed post-operative acupuncture treatment of canine surgical wounds, using a randomized, controlled and single blinded trial setup.

## Methods

After approval by the Institutional Research Ethical Committee and after all chosen dogs' owners had given their written consent, 29 otherwise clinically healthy dogs that were referred to surgery, were included into the study. Five dogs had two surgical wounds each (one of them underwent orthopaedic and soft tissue surgery during the same anesthesia). The health status of the dogs was confirmed by physical examination. Blood samples were collected from all the dogs and tested for hematocrit and urea values. These values were normal in dogs taken into the study. All surgeries were classified as class 1 in terms of contamination [20]. Contaminated wounds, like open fractures or surgeries at the anal area, were not included in the study. All surgeries were performed by an experienced surgeon blinded to the post-operative treatment. Different anesthesia protocols were used depending on the different kind of surgery conducted. All anesthesias were performed by a veterinary anaesthesiologist. To be able to evaluate the wound correctly, local anesthetics were not used at all.

The dogs were randomly and blindly divided into two wound treatment groups using paper pieces drawn from a hat. The randomization was stratified only for type of surgery (orthopaedic or soft tissue). The dogs were given the number(s) in the order they came in for the first visit. Appointment reservations were made by the hospital staff not knowing about the randomization list. 15 animals (five males and ten females, totally 17 wounds) were treated with dry needle acupuncture by a small animal veterinarian certified in veterinary acupuncture (by International Veterinary Acupuncture Society, IVAS). Treatment consisted of one acupuncture treatment right after the surgery, when all the animals were still under anesthesia, using the acupuncture points LI4, LI11, GB34, SP6, ST36, GV14 and two local points 0.5 cm distal from both ends of the wound. The size of the needles were 0.25 mm × 30 mm for dogs weighing above 10 kg and 0.20 mm × 15 mm for dogs weighing less than 10 kg (sterile Zhou acupuncture needles, Wuijiang Shenli Medical & Health Material C., Ltd). Sterile Han Il acupuncture 0.17 × 7 mm disposable needles (Han IL Acupuncture Needle Manufacturing Co.) were used for the local wound points. The needles were maintained in place for five minutes, except for the GV14 point, where the needle was maintained for 15 minutes. The control group consisted 14 cases (seven males and seven females, totally 17 wounds) that did not receive any post operative acupuncture treatment.

The median age of animals in the treatment group was 5 years (range 0.3-9.0) and in the control group 4.75 years (range 0.5-9.1). The median body weights (kg) were 7.7 (range 1.8-42.0) and 11.75 (range 2.1-43.0) and the median body condition scores [21] were 3/5 (range 2-5) and 3/5 (range 1-4) in the treatment and control groups, respectively. The duration of surgeries (hours) in the treatment group was (mean ± SE): 1.26 ± 0.23 and in the control group 1.19 ± 0.23. For more baseline information please see Table 1.

**Table 1:** Demographics of the dogs and their surgeries

**Dog ID**.	Breed	Age (years)	Sex	Weight (kg)	BCS	Type of surgery	Time (h)	Group (T/C)
**One wound per dog:**								

3	Boxer	5.6	M	39.4	4	Nodulectomy	0.75	T

6	Mixed breed	7	F	22.2	3	Mastectomy	0.75	T

8	Poodle	8.5	M	2.6	3	Diaphragmatic hernia	2.5	T

10	Cocker spaniel	6.9	F	17	5	Mastectomy	1.5	T

11	Pincher	9	F	3.3	3	Inguinal hernia	0.7	T

14	Mixed breed	5	M	6.2	3	Nefrectomy	1	T

15	Mixed breed	2	F	2.7	2	OHE	0.7	T

19	Mixed breed	3.8	F	8.4	3	OHE	0.5	C

20	Mixed breed	2.9	F	14	3	Nefrectomy + OHE	2	C

21	Rottweiler	9.1	M	43	3	Nodulectomy	0.5	C

22	Poodle	4	F	22	3	Nodulectomy	0.25	C

23	Mixed breed	1.1	F	7.7	3	OHE	0.5	T

25	Poodle	5.5	M	4	3	Femur osteosynthesis	1.5	C

27	Cocker spaniel	6.9	M	17.2	4	Patella fixation	1	C

28	Brazilian Fila	1.7	F	42	3	ACL	2	T

29	Mixed breed	5	F	5.9	3	Tibia osteosynthesis	1	T

30	Poodle	0.6	F	2.1	3	Femoral head amputation	0.5	C

31	Poodle	8.5	M	2.5	1	ACL	0.7	C

33	Yorkshire terrier	2.6	M	1.8	3	Patella fixation	1	T

34	Mixed breed	0.5	F	7.4	3	Femoral head amputation	1	C

37	Mixed breed	1.1	F	7.7	3	Femoral head amputation	0.4	T

38	Pitt bull	0.4	F	18.2	3	Pubic osteosynthesis	0.6	T

39	Rottweiler	8	M	11.7	3	Amputation of front leg	2.25	C

42	Mixed breed	2.5	M	8.6	4	Patella fixation	0.5	C

**Two wounds per dog:**								

4	Mixed breed	8	M	37.6	2	Nodulectomy and castration	3.5	T

12	Cocker spaniel	6.9	F	11.8	3	Nodulectomies, 2 sites	2.25	C

16	Pitt bull	2	F	25.5	3	OHE and ACL	3	C

17	Mixed breed	6	M	11.7	3	Inguinal hernia and castration	0.75	C

35	Pincher	0.3	F	2.4	3	Humerus osteosynthesis, 2 sides	2	T

Age (years), sex, weight, body condition score (BCS) [21], type of surgery and duration of surgery in dogs treated with acupuncture(T) or not (C). (OHE = ovarhysterectomy, ACL = anterior cruciate ligament repair, FHA = Femoral head amputation).

Standard disinfection was performed before and after the surgery with 0.5% Clorhexidine-solution (Riohex^®^, Indústria Farmacêutica Rioquimica Ltda) in all dogs. Single points skin sutures were performed with Nylon 2-0 or 3-0 (Shalon^®^, Shalon Fios Círurgicas Ltda). All owners received post operative care instructions including the use of Elisabethan collar and cleaning of the wound three times daily. A post-operative care table was also given to the owners, to be completed daily until removal of the stitches, seven days after surgery. Meloxicam 0.1 mg/kg SID was used as the only drug to treat post operative pain for five days, except in two animals that were further treated with oral tramadol (1.0-1.5 mg/kg TID) and three dogs that were treated with dipirone (25 mg/kg TID). Cephalexin 30 mg/kg BID was administered for seven days except in six cases, where enrofloxacine was used and in one case, where amoxicilline was used, due to surgeon's preference. Another veterinary surgeon, blinded to the treatments, evaluated the wounds at three and seven days after surgery. The first evaluation was performed at the owners' house and the last at the Veterinary Hospital before the stitches were removed. Evaluated variables included oedema (scale 0-3, where 0 = no oedema, 1 = little oedema, 2 = medium-grade oedema, 3 = massive oedema) scabs (yes/no), exudate (yes/no), haematoma (yes/no), dermatitis (yes/no), and humidity of the wound (dry/humid). All the assessments were done subjectively by the evaluating veterinarian. All wounds were photographed with a digital camera right after the surgery and at the time of the evaluations.

### Statistical analysis

A statistical power analysis based on a prior publication could not be performed, as no studies evaluating surgical wound treatment were found in dogs. A number of 17 dogs would be able to show a 45% difference of treatment effect, with 95% confidence level and 80% power. In statistical analyses each dog was a unit in the descriptive statistics whereas between or within groups each wound was a unit. Baseline bias and comparision of groups were analysed using a two-way independent t-tests, Mann-Whitney tests or Fisher's exact tests, depending on type of data. Most of the variables evaluated at day 3 and 7 were dichotomous and therefore not normally distributed. The Willcoxon Signed rank or McNemar's test was used for comparison between evaluations within each group. Differences were considered significant when the p value was less than 0.05.

## Results

**Figure 1:**
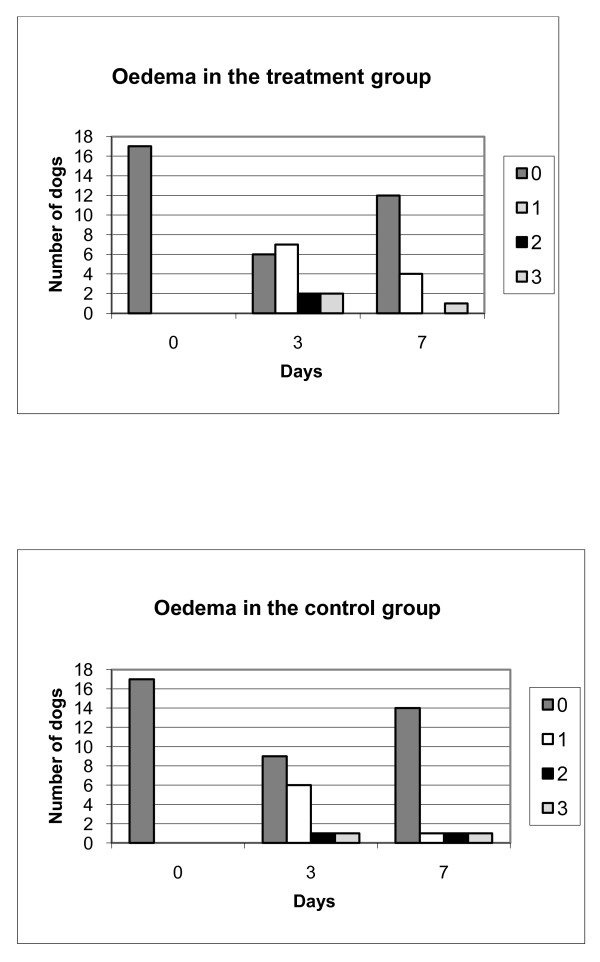
**Oedema of the wound in the two groups**. Oedema of the wound in the treatment and control group 0, 3 and 7 days after surgery. 0 = no oedema, 1 = little oedema, 2 = medium-grade oedema, 3 = massive oedema. n = 17 per group.

There was no baseline bias between the treatment and the control group in the following parameters: breed, age, sex, body condition score, type of surgery, duration of surgery, surgeon, chosen post operative antibiotic treatment and post operative wound size. There were no significant differences between the treatment and control groups in any of the variables evaluated three and seven days after surgery. In both treatment and control groups oedema increased significantly three days after surgery with some more oedema in the treatment group at day three, although the difference here was not significant between groups (Figure 1). The oedema decreased in both groups seven days after surgery. The reduction of the oedema was significant only in animals treated with acupuncture (p = 0.008). Scab formation increased in both groups between three to seven days after surgery, but this increase was not significant. There was more haematoma in the treatment group at three days after surgery and this difference was very close to being significant (p = 0.051) (Table 2).

**Table 2:** Wounds evaluated three and seven days after surgery, per group

		Scabs		Exudate		Hemathoma		Dermatitis		Humidity	
		
Group	Signs present	Day 3	Day 7	Day 3	Day 7	Day 3	Day 7	Day 3	Day 7	Day 3	Day 7
**Treatment**	**Yes**	4	9	4	0	4	2	2	0	3	1
	
	**No**	13	8	13	17	13	15	15	17	14	16

**Control**	**Yes**	6	11	1	1	0	0	1	0	1	2
	
	**No**	11	6	16	16	17	17	16	17	16	15

The same person evaluated the wounds three and seven days after surgery and scored five variables as being present or not. There were no significant differences of these variables between groups or within groups, between evaluations at 3 and 7 days (p < 0.05). n = 17 per group.

## Discussion

One dry needle acupuncture treatment in dogs using acupuncture points LI4, LI11, GB34, SP6, ST36, GV14 and two local points 0.5 cm distal from both ends of the wound right after soft tissue and orthopaedic surgery maybe decreased post-operative oedema faster. Similar results have been reported before in studies where oedema was induced in experimental animals [8,12,14]. However, when considering these results, it must be noted that there also were more dogs with oedema in the treatment group three days after the surgery, even if this difference between groups was not significant.

For years the only sham treatment allowed in acupuncture trials has been insertion of needles in non-acupuncture points. In this study, the dogs of the control group did not receive sham acupuncture. This is important, as it lately has been reported that sham acupuncture produces similar, although often less pronounced, effects than real acupuncture [22-29]. Therefore sham acupuncture should never be used as a placebo treatment [25,26,28]. However, in trials where an acupuncture treatment has been compared to a non-treated group (e.g. waiting list groups), significant differences have been reported [30,31]. As we did not expect our dogs to be able to anticipate neither an upcoming post-operative treatment, nor if it was not performed, we felt it was ok to just leave the other group without treatment. The fact that all dogs were acupuncture naïve and still under residual anesthesia further strengthened this assumption. In canine studies dogs are not likely to get a positive placebo response only from the fact that they have been treated or a negative "nocebo" response from not having been treated, as humans would do. Therefore, a single blind study, where the dog, the owner and the medical personnel evaluating the treatment effect were blinded, was considered a good trial design.

The first limitation of this study is the difficulty to objectively evaluate wound healing. A tensiometer has been used in experimental wound studies [32,33], but this has not been validated for different dog breeds and also it was not available for us. As dog size ranged from 2 to 43 kg, it is impossible to measure scar tissue or oedema with a millimeter measure, as they partly will be proportional to dog size. The method used by us has been reported by Han et al [34]; they used a scale of 0-3 for oedema and redness in their experimental study of wound healing in rats.

Another major limitation was that the wounds evaluated in this study varied in size and location as different types of surgeries were included. The mechanical stress of the wound is one of the local factors that influence surgical wound healing [3]; according to that, selection of only one type of surgery resulting in wounds of similar length and place would have been ideal. However, most of the soft tissue surgeries were performed on the main body and most of the orthopaedic surgeries on the limbs. The duration of the surgical procedure was also very variable, which could have an impact on the wound healing. Although the owners' postoperative care table indicated that the home care had been similar in all dogs, differences in environment and owners care cannot be disregarded either.

Other limitations include the relatively small number of dogs per group and heterogeneous cases with respect to age and body condition score. Although animals more than 10 years old were excluded from the study, there was still a large variation between animals. It is considered that wounds of young animals heal better than those of older animals and underweight and malnutrition can also influence the wound healing process [3]. However, there was no significant difference between treatment and control groups in either of these variables. As this was a pilot study, 17 dogs per group should have been enough.

The last limitation was the fact that patients only got one single acupuncture treatment. As the aim was to find an easy protocol that would not require any extra visits, one single treatment was all that was tested. Knowing that acupuncture treatments are usually always given as a minimum of three times [4], it is very possible that also this had an impact on the results. It would have been interesting to see if more treatments would have strengthened these quite weak positive results.

All these issues should be addressed in future studies.

## Conclusions

As a conclusion, in our study one dry needle acupuncture treatment performed right after surgery did not seem to have any immediate effect on wound healing although a significant decrease in oedema and an increase in haematomas could be seen within the treatment group. As other researchers previously have found acupuncture to fasten wound healing, we hope this pilot study will inspire further studies on this topic.

## Competing interests

The authors declare that they have no competing interests.

## Authors' contributions

EES participated in the design of the study, did (or did not) the acupuncture treatments on the dogs, performed part of the statistical analysis and wrote the article. AKH-B performed major part of the statistical analysis and helped to draft the manuscript. KH participated in the design of the study and evaluating the wounds. EKK and CVSB participated in the design of the study. SPLL did the major part of the design of the study and helped with the statistical analysis and to draft the manuscript. All authors read and approved the final manuscript.

## Authors' information

EES (DVM) was doing part of her post graduation program studies at the School of Veterinary Medicine and Animal Science of São Paulo State University in Brazil as an exchange post graduate student. She is also a Certified Veterinary Acupuncturist (CVA, by the International Veterinary Acupuncture Society). This study is part of her post graduation program. Nowadays she is working in a private small animal practice in Finland. AKH-B (DVM, PhD, CVA) is working at the Faculty of Veterinary Medicine, Department of Equine and Small Animal Medicine, University of Helsinki as a researcher and acupuncturist. KH (DVM) was doing her residence program in surgery in the School of Veterinary Medicine and Animal Science of São Paulo State University in Brazil at the time of the study. EKK (DVM, PhD) is working in Faculty of Veterinary Medicine, Department of Equine and Small Animal Medicine in University of Helsinki as a clinical teacher. She is also mentor of EES's post graduation. CVSB (DVM, Phd) is working at the Department of Veterinary Surgery and Anaesthesiology of the School of Veterinary Medicine and Animal Science of São Paulo State University in Brazil as a professor of surgery. SPLL (DVM, Phd, Dipl. ECVA, CVA) is working at the Department of Veterinary Surgery and Anaesthesiology of the School of Veterinary Medicine and Animal Science of São Paulo State University in Brazil as a professor of anaesthesiology. He was also the local mentor of EES's post graduation program during the exchange.
